# Editorial: Overcoming the Immune Microenvironment of Hepatocellular Cancer

**DOI:** 10.3389/fimmu.2021.707329

**Published:** 2021-06-02

**Authors:** Shishir Shetty, Amaija Lujambio, Frank Tacke

**Affiliations:** ^1^ Centre for Liver and Gastrointestinal Research, Institute of Immunology and Immunotherapy, Medical School, University of Birmingham, Birmingham, United Kingdom; ^2^ Department of Oncological Sciences, Tisch Cancer Institute, Icahn School of Medicine at Mount Sinai, New York, NY, United States; ^3^ Department of Hepatology & Gastroenterology, Charité - Universitätsmedizin Berlin, Berlin, Germany

**Keywords:** hepatocellular carcinoma, immunotherapy, tumour microenvironment, chronic liver disease, fibrosis

Hepatocellular carcinoma (HCC) is a leading cause of global cancer-related deaths and cases are predicted to rise in the coming decades. In the vast majority of patients, HCC develops in the context of chronic liver disease, which provides the setting for a complex tumour microenvironment characterised by constant induction of cell death with compensatory hyperproliferation, chronic inflammation, maladaptive wound healing, and fibrosis. While this inflammatory microenvironment provides an overall pro-carcinogenic milieu, the hepatic immune system also participates in tumour surveillance and anti-tumour immune responses. Targeting the tumour microenvironment is therefore a critical strategy in both treating advanced HCC and preventing tumour recurrence in patients undergoing curative therapies. The recent approval for the use of immunotherapy for treating HCC, specifically the combination of immune checkpoint blockers with anti-VEGF agents, has helped to confirm the long held belief that targeting the immune microenvironment could be an effective approach to treating this tumour. While immuno-oncological therapeutic options generally provided survival benefit for advanced, non-resectable HCC with manageable side effects compared to previous medical approaches, treatment efficacy is still not satisfying and patient stratification is not well defined. The aim of this article collection is to highlight new pathways that may help in developing novel immunotherapeutic approaches for HCC and to explore the optimal use of immunotherapies in the context of the expanding arsenal of therapies that are now becoming available for advanced HCC.

## Exploring How Tumour Cells Drive the Immune Microenvironment in HCC

The last few years have seen a major interest in the study of the tumour microenvironment (TME). It is now clear that tumours not only have intrinsic effects that promote cell survival/proliferation but can also influence the extrinsic tissue microenvironment and drive a programme of immune evasion. Lu et al. report that the Na+/K+-ATPase, ATP1B3, is upregulated in HCC tissue and HCC cell lines and proteomic analysis of publically available databases confirmed a correlation with tumour immune infiltrates. Further functional assays demonstrate that ATP1B3 contributes to key tumourigenic pathways including cell proliferation and migration, and raise the possibility of targeting ATP1B3 in patients with HCC. In another article, Jiang et al. focus on the contribution of Tank-binding kinase 1 (TBK1) in regulating CD8 T cell infiltration in HCC. Using a model of HCC developed on the background of chronic liver inflammation, they demonstrate that an inhibitor of TBK1 alters the cytokine milieu within the tumour and promotes CD8 T cell infiltration leading to suppression of tumour growth. The need to take into account other characteristics of the tumour microenvironment, specifically hypoxia, is highlighted by the analysis carried out by Mo et al. Based on large HCC genomic datasets, they identified a link between hypoxia-associated genes and immunosuppressive features in tumour samples. The hypoxia gene signature may be a potential tool for stratifying patients for immunotherapy but also sheds light on the need to overcome alternative TME pathways in order to boost the efficacy of immunotherapy.

## Boosting Communication in the HCC Microenvironment

Whilst T cells are the critical effector arm of the immune system in attacking and preventing tumours, they are educated by a range of innate and adaptive immune populations. In the context of HCC, the liver microenvironment itself has a vital role in influencing T cell function by way of its resident non-parenchymal cell populations including Kupffer cells, liver sinusoidal endothelial cells, and hepatic stellate cells. In their review detailing the landscape of HCC, Giraud et al. cover the factors that initiate tumour development in the liver and subsequent tumour progression. They highlight the complex cross-talk between the immune system and hepatic microenvironment and summarise the clinical trials that are now taking place building on the knowledge that has been gathered in the field. Lurje et al. focus on the critical role of antigen presentation in the cross-talk between immune cells and how promoting effective antigen presentation could switch the TME from an immunosuppressive to an immunostimulatory state. They review the rationale for *in situ* vaccination and cover the mechanisms of antigen presentation and the range of approaches that are being undertaken to harness the TME.

## Immunotherapy as the New Standard of Care for HCC

The advent of immunotherapy has opened up new options for patients with HCC, yet the outlook in patients with advanced disease remains poor and there still remain significant questions regarding the stratification of patients for immunotherapy. Sharma and Motedayen Aval highlight the issues surrounding second line agents in patients with advanced HCC. They provide a summary of approaches being considered for novel combination therapies to overcome the resistance to – or lack of efficacy – of immune checkpoint inhibitors. Looking at the experience with renal cell carcinoma they also discuss the sequential use of Tyrosine Kinase inhibitors following immunotherapy. In another article, Mohr et al. review the journey towards the use of immune checkpoint inhibitors in HCC but also highlight the questions that still need answering regarding the optimal use of these therapies in the setting of current treatment algorithms. Such open questions include optimal treatment options in non-viral induced HCC or for patients with advanced stage cirrhosis (e.g. Child B or C). Moreover, they summarise the future directions with immune checkpoint inhibitors including combinations with novel immunotherapies as well as elucidating the role of immunotherapy in the neoadjuvant setting and combining with loco-regional therapy and trans-arterial chemoembolization.

Immunotherapy has been an exciting breakthrough in HCC yet the tumour microenvironment of HCC still remains a major challenge to therapeutic success. Understanding the optimal use of immunotherapies in the clinical setting and the identification of new therapies to boost the efficacy of current strategies will hopefully lead to major improvements in survival for patients with HCC.

## Author Contributions

All authors made a contribution to writing the editorial and approved it for publication.

## Funding

SS is funded by a Cancer Research UK Advanced Clinician Scientist fellowship C53575/A29959 and supported by HUNTER funded through a partnership between Cancer Research United Kingdom, Fondazione AIRC and Fundación Científica de la Asociación Española Contra el Cáncer. AL is funded by NIH/NCI R37CA230636 and Damon Runyon-Rachleff Innovator Award.

## Conflict of Interest

SS receives consultancy fees from Faron Pharmaceuticals. Work in the lab of FT has received funding by Allergan, Inventiva, Gilead and BMS. Work in the lab of AL has received funding from Pfizer and Genentech for unrelated projects.

